# OCCUPATIONAL PHYSICAL HAZARDS AND RISK FOR DEMENTIA AND MILD COGNITIVE IMPAIRMENT IN THE HUNT 70+ STUDY

**DOI:** 10.1093/geroni/igae098.0973

**Published:** 2024-12-31

**Authors:** Sarah Tom, Bernt Bratsberg, Bo Engdahl, Yaakov Stern, Hans-Peter Kohler, Asta Håberg, Bjørn Heine Strand, Vegard Skirbekk

**Affiliations:** Columbia University, New York, New York, United States; Ragnar Frisch Centre for Economic Research, Oslo, Oslo, Norway; Norwegian Institute of Public Health, Oslo, Oslo, Norway; Columbia University, New York, New York, United States; University of Pennsylvania, Philadelphia, Pennsylvania, United States; Norwegian University of Science and Technology, Trondheim, Sor-Trondelag, Norway; Norwegian Institute of Public Health, Oslo, Oslo, Norway; Norwegian Institute of Public Health, Oslo, Oslo, Norway

## Abstract

**Background:**

While occupational physical hazards are related to physical decline in older adulthood, less is known about cognitive outcomes. We hypothesize that greater life course occupational physical hazards would be associated with higher mild cognitive impairment (MCI) and dementia risk.

**Methods:**

Data were from the Trøndelag Health Study, a population-based study of one Norwegian county beginning in 1984, with follow-up every decade. In 2017-2019 participants age ≥ 70 had cognitive assessments, used to diagnose MCI or dementia. Occupations from age 30-65 were from decennial censuses (1960 - 1980) and the national employer-employee register, from 1995. Occupations were assigned to International Standard Classification of Occupation-88 codes, linked to the Occupational Information Network. Occupational physical hazards score is the mean of 10 characteristics describing exposures to hazardous and adverse occupational environmental conditions. Age, sex, marital status at age 30, and education were from registers. Ordered logistic regression models assessed the relationship between occupational physical hazards and later-life cognitive status.

**Results:**

In 9344 participants median occupational physical hazards score was 1.82 (range: 1.0-3.3 (greater hazard)). The mean age was 78, and 35% and 15% of participants had MCI and dementia diagnoses, respectively. Adjusting for age and sex, one standard deviation greater occupational physical hazards were related to 1.32 times the risk of worse cognitive level (OR 1.32, 95% CI 1.26-1.36). Adjusting for education and marital status attenuated this association (OR 1.16, 95% CI 1.11-1.22).

**Conclusions:**

Those exposed to occupational physical hazards may need greater support with later life MCI and dementia risk.

